# Postoperative antibiotic treatment does not lower re-revision rate in presumed aseptic hip and knee revision arthroplasties with unexpected positive intraoperative cultures – a matched cohort study

**DOI:** 10.5194/jbji-10-51-2025

**Published:** 2025-03-07

**Authors:** Sebastian Simon, Marjan Wouthuyzen-Bakker, Susana Gardete Hartmann, Jennyfer A. Mitterer, Sujeesh Sebastian, Stephanie Huber, Bernhard J. H. Frank, Jochen G. Hofstaetter

**Affiliations:** 1 Michael Ogon Laboratory for Orthopaedic Research, Orthopaedic Hospital Vienna-Speising, 1130 Vienna, Austria; 2 AUVA Trauma Center Meidling, 1120 Vienna, Austria; 3 Department of Medical Microbiology and Infection Prevention, University of Groningen, University Medical Center Groningen, Groningen, the Netherlands; 4 2nd Department of Orthopaedic Surgery, Orthopaedic Hospital Vienna-Speising, 1130 Vienna, Austria; 5 AUVA Trauma hospital Lorenz Boehler, 1200 Vienna, Austria

## Abstract

**Aims**: It remains unclear if postoperative antibiotic (AB) treatment is advantageous in presumed aseptic revision arthroplasties of the hip (rTHA) and knee (rTKA) with unexpected positive intraoperative cultures (UPIC). The aim of this study is to evaluate if there is a difference in the re-revision rate in patients with UPIC when treated with postoperative AB or when postoperative AB is withheld. **Methods**: In this retrospective matched cohort study we compared the re-revision rates in rTHA and rTKA with (AB group: 45 rTHA, 25 rTKA) and without (non-AB group: 45 rTHA, 25 rTKA) AB treatment in patients with UPIC. Baseline covariates for matching were the microorganism (likely or not likely to be a contaminant), patient demographics, joint, revision type, surgical site infection score, American Society of Anesthesiologists classification, serum C-reactive protein (CRP). **Results**: After a median follow-up of 4.1 (inter-quartile range, IQR: 2.9–5.5) years after rTHA and rTKA, the re-revision rate between the AB group and the non-AB group was 14.3 % versus 15.7 % (*P*=0.81). In the AB group, 4.3 % (3/70) of patients underwent revision due to septic complications compared to 5.7 % (4/70) in the non-AB group (*P*=0.69). None of the patients were diagnosed with a confirmed periprosthetic joint infection (PJI) according to the PJI diagnostic criteria of European Bone and Joint Infection Society (EBJIS). In 22/70 (31.4 %) of the patients in the AB group and in 15/70 (21.4 %) of the patients in the non-AB group, a diagnosis of “infection likely” was made according to the EBJIS criteria (*P*=0.18). All UPICs with low virulent microorganisms were considered to be contamination (coagulase-negative *Staphylococci*; *Corynebacterium*; anaerobic Gram-positive bacilli and cocci, e.g., *Finegoldia magna*, *Cutibacterium acnes*). **Conclusion**: Postoperative AB treatment did not result in a decreased re-revision rate in patients with UPIC in presumed aseptic rTHA and rTKA. Patients diagnosed with pathogens classified as a likely contaminant can be safely ignored.

## 1 Introduction

Loosening of a prosthetic joint is one of the most common indications for hip and knee revision surgery (Kenney et al., 2019; Di Martino et al., 2021; Postler et al., 2018). Unexpected positive intraoperative culture (UPIC) in presumed aseptic revision total hip (rTHA) and knee (rTKA) arthroplasty is described in between 5.9 % and 38 % of cases (Barrack et al., 2007; Jacobs et al., 2017; Ribera et al., 2014).

UPICs are often associated with low-virulent pathogens and single positive cultures, which can be due to contamination (Goh et al., 2022; Hipfl et al., 2021; Simon et al., 2023). It is well established that microorganisms can live on or around implants without signs or symptoms of infection or they cause no harm but can lead to loosening (Jakobsen et al., 2018; Parvizi and Gehrke, 2018). Antibiotic treatment is essential if a periprosthetic joint infection (PJI) is diagnosed (Miller et al., 2020). However, the clinical relevance of microbiological findings in presumed aseptic revisions is not entirely clear, and treatment recommendations differ (Goh et al., 2022; Kloos et al., 2022). There is inconclusive evidence on whether antibiotics (ABs) should be started in patients with UPIC when infection was not suspected during the preoperative diagnostic workup (Goh et al., 2022; Saleh et al., 2014). Furthermore, the current literature regarding the association between UPICs and revision rates is also inconclusive. While some studies have reported a higher revision rate in patients with UPICs (Milandt et al., 2019; Schwarze et al., 2022; Staats et al., 2017; Vargas-Reverón et al., 2025), others have not identified a significant difference in this regard (Goh et al., 2022; Neufeld et al., 2021, 2022).

The 2018 International Consensus Meeting on Musculoskeletal Infection (ICM) provides limited evidence regarding the administration of AB therapy to patients with UPIC (Parvizi and Gehrke, 2018). Differences between proposed treatment algorithms in UPIC may be due to small sample sizes and the heterogeneity of patients included in studies (Purudappa et al., 2020). The European Bone and Joint Infection Society (EBJIS) definition of 2021 states that a PJI classified as infection likely does not require AB treatment if the pathogen is likely to be a contaminant (McNally et al., 2021). However, to date, there is very little to no evidence on this topic (Wu et al., 2024).

The aim of this study is to assess whether the administration of postoperative ABs is associated with a reduced incidence of septic and/or aseptic re-revision in THA and TKA with UPIC in presumed aseptic revisions.

## 2 Methods

### 2.1 Methods

This retrospective matched cohort study was approved by the institutional review board (EK11/2020). We analyzed our institutional arthroplasty registry and prospectively maintained the PJI database between 1 January 2011 and 31 December 2020. The study included presumed aseptic knee and hip revisions with a minimum follow-up of 2 years.

Presumed aseptic revisions were defined as revisions with no clinical signs of infection and no or one preoperative positive criteria according to the EBJIS infection likely group. For rTHA, single-stage replacements, one- or two-component replacements, and head and liner replacements for aseptic reasons were included. For rTKA, only single-stage replacements for aseptic loosening were included. Revisions were excluded if (1) PJI was known or suspected preoperatively, (2) the revision was part of the management of an ongoing PJI (second stage of a two-stage revision), or (3) no intraoperative cultures were obtained or results were not available.

The patient's demographic data, surgical site infections (SSIs), and the reason for revision and re-revision were evaluated (Austin, 2011; Everhart et al., 2016; Parvizi et al., 2018). Tissue samples or swabs were taken intraoperatively in all included patients, and explanted devices were put into sonication containers and processed as described before (Frank et al., 2021).

Patients with presumed aseptic loosening and UPIC were postoperatively diagnosed according to the PJI definition of the EBJIS into the categories of infection unlikely, infection likely, and infection confirmed. Additionally, the causative pathogen(s), knee or hip joint, and revision following primary or revision surgery were analyzed. Pathogens were classified into two categories: those likely to be contaminants and those not likely to be contaminants (Sousa et al., 2023). The following microorganisms were not likely to be contaminants: *Staphylococcus aureus*, *Staphylococcus lugdunensis*, beta-hemolytic streptococci, *Streptococcus anginosus* group, enterococci, Enterobacterales, *Pseudomonas aeruginosa*, anaerobic Gram-negative rods, and *Candida*. The following microorganisms were likely to be contaminants: most coagulase-negative staphylococci (*S. epidermidis*, *S. capitis*, *S. hominis*, *S. warneri*, or *S. haemolyticus*), micrococci, *Corynebacterium*, anaerobic Gram-positive bacilli (*Cutibacterium acnes*), anaerobic Gram-positive cocci (*Finegoldia magna*, *Acinetobacter lwoffii*, and *Neisseria* spp.).

### 2.2 Study cohorts according to AB treatment

All patients received routine intravenous (IV) first-generation cephalosporin (or vancomycin for those with a history of allergy to penicillin or cephalosporins) prior to surgical incision as antibiotic prophylaxis. Postoperative empirical AB treatment was decided on an individual basis according to surgeon and infectious disease specialist preference. Patients were subsequently divided into two groups.

1. *AB group*. The AB group received intravenous or per os postoperative AB treatment within 4 d postoperatively with a minimum duration of 2 weeks. Empirical AB treatment was based on our institutional guidelines, and once microbiological test results were available, the regimen was changed according to the recommendations of our infectious disease specialist.
2. *Non-AB group*. The non-AB group received perioperative prophylaxis with cephalosporin or vancomycin but no further postoperative AB treatment.

**Figure 1:**
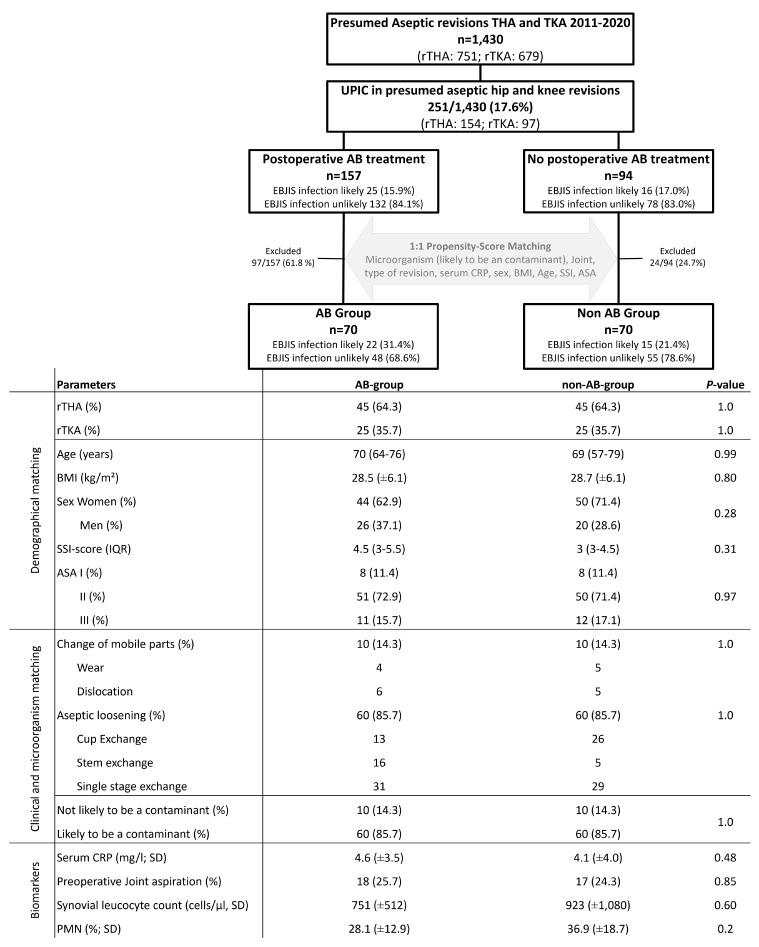
**Figure 1**Flow chart for propensity score matching in rTHA (revision total hip arthroplasty) and rTKA (revision total knee arthroplasty) with UPICs (unexpected positive intraoperative cultures). EBJIS (European Bone and Joint Infection Society), BMI (body mass index), SSI (surgical site infections), AB (antibiotic), PMN (polymorphonuclear leukocytes), ASA (American Society of Anesthesiologists Classification), and median with IQR (inter-quartile range).

A matching between the UPIC patients treated with antibiotics (AB group) and the patient treated without antibiotics (non-AB group) was performed using defined baseline covariates. The microorganism (likely or not likely to be a contaminant), joint (hip/knee), reason for revision, serum CRP (C-reactive protein), sex, BMI (body mass index), age, ASA (American Society of Anesthesiologists), and SSI score (0–35) (Everhart et al., 2016) were baseline covariates to conduct a 1:1 matching via z scoring (Fig. 1). A z score was created for each of these covariates to create one total z score including all covariates. The most similar z score between one patient from the AB group was matched with one patient from the non-AB group (Fig. 1). In total, 140 rTHA and rTKA (70 in the AB group, 70 in the non-AB group) patients were included in the study. There was no significant distribution of patient demographics or preoperative PJI workup in either rTHA or rTKA (Fig. 1).

The outcome of patients with UPICs treated with ABs and without ABs was analyzed by evaluating the septic and aseptic re-revision rates during follow-up. A septic re-revision rate was defined as a confirmed PJI according to the EBJIS definition. The minimum follow-up period was 24 months. Follow-up was conducted through patient recall and a review of the clinical databases for clinical visits.

### 2.3 Statistical analyses

Descriptive statistics were used with means (M), standard deviations (SD) and medians (Md) for continuous study parameters and frequencies and percentages for categorical variables. If the data were skewed, we used the interquartile range (IQR). Continuous data were compared using Mann–Whitney *U* tests or two-sample *t* tests for non-parametric and parametric data, respectively. Categorical data were compared using Pearson's *χ*^2^ test or Fisher's exact test as appropriate. The Kaplan–Meier method with 95 % confidence intervals (CIs) was used to determine revision-free implant survival at 1, 2, 5, and 8 years for the AB group and the non-AB group with subsequent septic or aseptic revision as the end point. Patients who died or were lost to follow-up after 2 years were censored. The 95 % CIs were calculated using the Greenwood asymmetric exponential formula. Statistical significance was two-tailed and set at a *P* value ≤ 0.05. All analyses were performed with IBM SPSS^®^ version 25 and GraphPad Prism 8.

## 3 Results

### 3.1 Results

**Figure 2:**
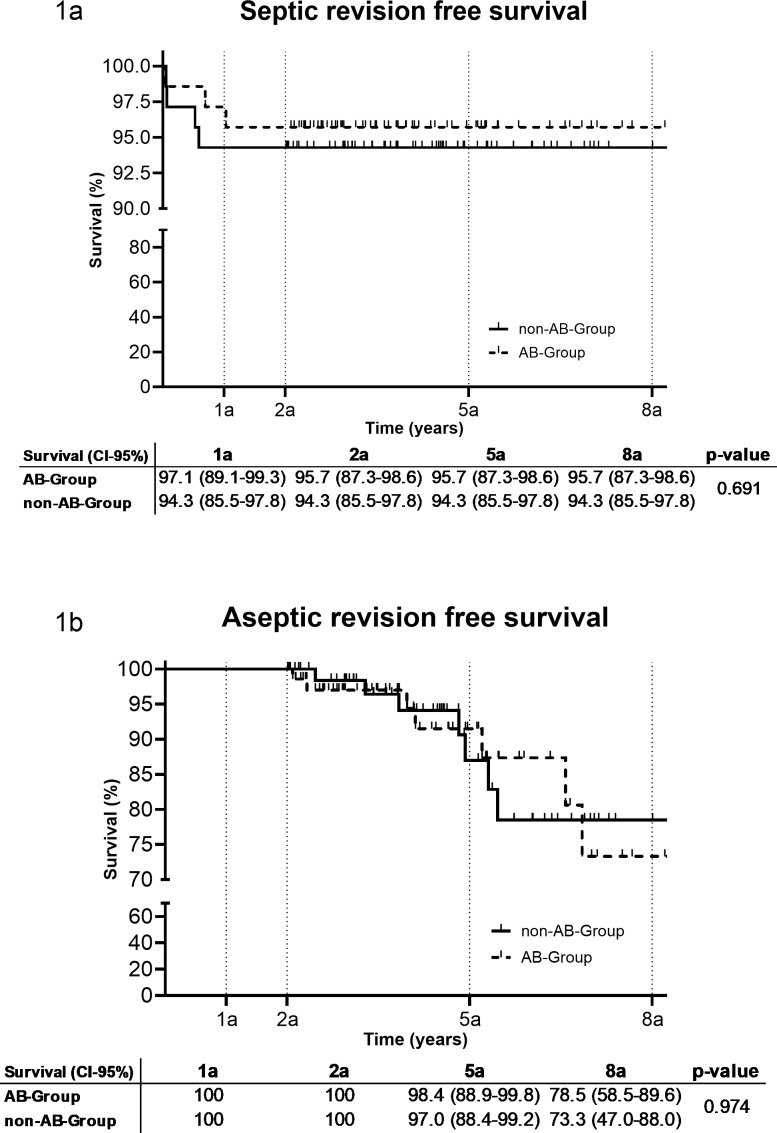
**Figure 2**Revision-free implant survival after 1 a, 2 a, 5 a and 8 a (95 % confidence interval) (a: years, AB: antibiotic).

After a median follow-up of 4.1 years (IQR: 2.9–5.5), the re-revision rate between the AB group (10/70, 14.3 %) and the non-AB group (11/70, 15.7 %) was similar (*P*=0.81). In the AB group, three patients were revised due to septic reasons after 13, 253, and 376 d, while seven were revised for aseptic reasons (four dislocations and one hematoma, one wear, one loosening). In the non-AB group, four patients were revised due to septic reasons after 18, 24, 192, and 214 d, while seven were revised for aseptic reasons (three dislocations, three loosening, and one wear). There was no difference in the septic or aseptic revision rate between the AB group and the non-AB group after 1, 2, 5, and 8 years (Fig. 2). In two out of three (66.7 %) septic re-revisions in the AB group and in four out of four (100 %) septic re-revisions in the non-AB group, intraoperative tissue cultures were positive. The same microorganism, as during the initial revision, was identified in two out of four in the non-AB group and in one out of three in the AB group but all with different antibiograms. One additional microorganism was found in the AB group. In the infection likely group, 2 out of 37 (5.4 %) patients had a septic re-revision during follow-up compared to 5 out of 103 (4.9 %) in the infection unlikely group (*P*>0.99).

Overall, 65/70 (92.9 %) patients in the AB group received AB treatment with at least two different ABs. In total, 57/70 (84.4 %) patients received IV and oral ABs, 8/70 (11.4 %) received two oral ABs, 3/70 (4.3 %) patients received one IV AB, and 2/70 (2.9 %) patients were treated with only one oral AB. The mean duration of IV treatment was 9 d (IQR: 6–14). The total duration of AB treatment was 41 d (IQR: 23.5–56.5).

### 3.2 PJI diagnosis and microbiological spectrum of UPICs

According to the EBJIS definition, none of the patients with UPIC were classified as a confirmed PJI. However, 22 out of 70 (31.4 %) in the AB group, and 15 out of 70 (21.4 %) in the non-AB group were classified as infection likely (*P*=0.18). All patients in the infection likely category had an elevated serum CRP level between 10 and 20 mg L^−1^ and at least one positive intraoperative culture. In total, 7 out of 35 (20.0 %) patients had an increased leucocyte count between 1500 and 3000 cells per µL in synovial fluid. A total of 140 UPICs were evaluated in rTHA and rTKA procedures. All UPICs with low virulent microorganisms were considered a likely contaminant.

The results of all intraoperative microbiological analyses are presented in Table 1. The mean number of intraoperative cultures was 3.4 (±2.4). None of the patients had two positive samples with the same microorganism. Sonication was performed in 16/70 (22.9 %) of patients in the AB group and in 7/70 (10.0 %) of patients in the non-AB group. None of the patients had a positive sonication fluid culture according to the 50 colony forming units (CFU) per mL cut-off (McNally et al., 2021).

**Table 1:**
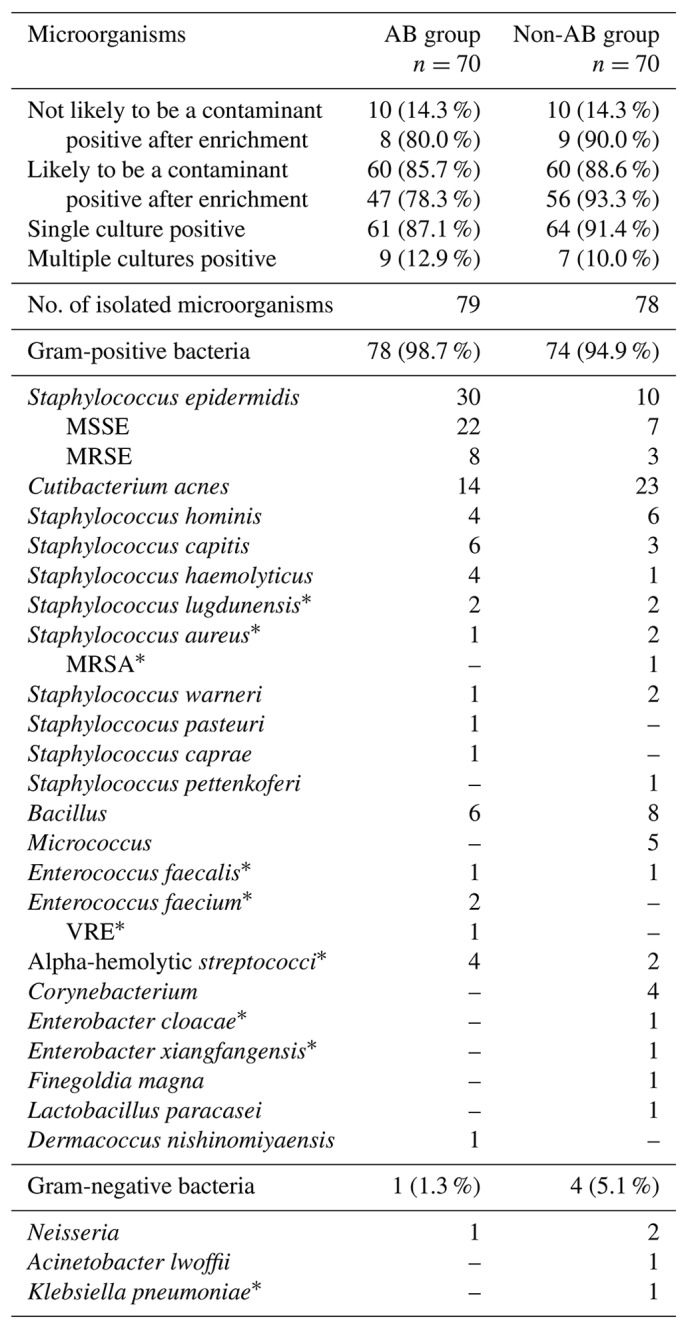
**Table 1**Microbiological spectrum for unexpected positive intraoperative cultures in revision total knee and hip arthroplasties in the AB group and the non-AB group. MSSE (methicillin-susceptible *Staphylococcus epidermidis*); MRSE (methicillin-resistant *Staphylococcus epidermidis*), MRSA (methicillin-resistant *Staphylococcus aureus*), VRE (vancomycin resistant enterococcus). ^*^ Not likely to be a contaminant.

A total of 25 distinct microorganisms were identified from intraoperative cultures. The most prevalent microorganisms were *Staphylococcus epidermidis* (25.5 %), *Cutibacterium acnes* (23.6 %) and coagulase-negative staphylococci (23.6 %). The number of *S. epidermidis* cultures was higher in the AB group (30/70 (42.9 %)) compared to the non-AB group (10/70 (14.3 %); *P*<0.001). There were more positive cultures after enrichment in the non-AB group (65/70 (92.9 %)) compared to the AB group (55/70 (78.6 %); *P*=0.02).

## 4 Discussion

This matched single-center cohort study demonstrated that postoperative AB treatment did not result in a lower septic or aseptic re-revision rate during follow-up in patients who underwent a presumed aseptic hip or knee revision with an UPIC in conventional microbiology.

There is clear consensus in favor of AB treatment after septic revision surgery when two or more intraoperative cultures isolate the same microorganism (Le Vavasseur and Zeller, 2022). However, controversy exists regarding the necessity of treating UPICs with ABs in cases with presumed aseptic loosening (Izakovicova et al., 2019; Wu et al., 2024). Various approaches have been suggested, including observation of the situation for a period of time, intravenous AB treatment for a certain duration, or long-term antibiotic suppression (Fernandez-Sampedro et al., 2015; Goh et al., 2022; Kloos et al., 2022; Purudappa et al., 2020; Saleh et al., 2014). ABs may not be required when only a single intraoperative culture identifies a microorganism. However, there may be circumstances when a single positive culture may indicate treatment (Parvizi and Gehrke, 2018). According to the EBJIS definition, an infection is considered likely in single positive cultures (McNally et al., 2021). However, the clinical relevance of a single positive culture of a common contaminant is debatable, and it is not clear whether this requires AB treatment (McNally et al., 2021).

On the one hand, AB therapy may not be necessary if a microorganism is isolated from a single intraoperative culture (Barrack et al., 2007). A recent study by Goh et al. (2022) suggested that positive cultures can be safely ignored in revision arthroplasty patients who do not meet the ICM-2018 criteria for PJI as long as these patients are appropriately investigated preoperatively. In their study, no patient received prolonged oral ABs after revision surgery. In comparison to the culture-negative group, the culture-positive group had no significant difference in the overall re-revision rate (Goh et al., 2022). On the other hand, Saleh et al. (2014) argued that PJI cannot be ruled out in patients with a single positive culture, particularly when a virulent organism is isolated or in the presence of other signs of infection. This study demonstrated a higher recurrence rate of infection in patients with a single positive intraoperative culture than in those with a negative culture (Saleh et al., 2014). In comparison to Saleh et al. (2014), we did not observe a higher re-revision rate for pathogens that are uncommon contaminants. However, the number of patients was insufficient to conclude that no treatment is necessary.

The total septic re-revision was slightly higher in this study compared to the study by Goh et al. (2022). This could be explained by the fact that only UPICs and no culture-negative revisions were included in our study. However, the septic re-revision rate of our study was lower compared to the study by Saleh et al. (2014). The lack of AB treatment did not result in a higher overall revision rate in UPICs even in patients with microorganisms that were uncommon contaminants. Consequently, our results are consistent with those of Goh et al. (2022).

However, there may be circumstances where a UPIC single positive culture, in combination with other positive diagnostic tests, may indicate the presence of infection, and treatment would be indicated. This decision should always be made by a multidisciplinary team, taking into account all available diagnostic tools and preoperative PJI workup (Mitterer et al., 2023). In this study, experienced arthroplasty surgeons, in collaboration with an infectious disease specialist, made decisions on a case-by-case basis, which resulted in a low rate of septic failure.

In some patients in this study, serum CRP levels were slightly elevated preoperatively, but there were no other signs of infection or other elevated parameters to confirm PJI, and therefore the patients were considered aseptic. In the study by Akgün et al. (2018), serum CRP levels alone cannot be used to confirm or exclude PJI and verify between specific pathogens and types of infections (Akgün et al., 2018). In patients with single positive UPICs with pathogenes likely to be contaminants and slightly elevated serum CRP levels, it is often difficult to decide whether the microbiological findings should be ignored or not. However, in this study, these patients did not have a significantly higher septic re-revision.

The matching performed in this study minimized the limitations of the retrospective design. Although the most important co-founders were included in the propensity score matching, not all possible co-founders could be included due to the high heterogeneity of the patients. Therefore, there is a potential selection bias. In addition, the decision to treat with ABs introduces an inherent selection bias into the study. There were no specific patient characteristics for treatment or no treatment as there were no guidelines for treatment, and the decision was made on an individual basis. Antibiotic treatment was heterogeneous between patients with the inclusion criteria for AB treatment; in this study, we try to perform a more homogeneous group by defining a minimum antibiotic treatment period. Compliance was not measured in patients receiving AB treatment. Moreover, not all patients received a full preoperative assessment, which may have resulted in missed PJI diagnoses.

## 5 Conclusion

In conclusion, AB treatment of UPIC in patients undergoing rTHA and rTKA did not result in a lower re-revision rate during follow-up compared to untreated cases. It should be noted that this cohort comprised patients in which no confirmed PJI was diagnosed and that the patients who were diagnosed as having an infection likely all had UPIC with microorganisms that are common contaminants.

## Data Availability

All raw data can be provided upon reasonable request from the corresponding author. Data are located in a controlled access data storage at the Michael Ogon Laboratory for Orthopaedic Research, Orthopaedic Hospital Vienna-Speising.
